# Curcumin inhibited hepatitis B viral entry through NTCP binding

**DOI:** 10.1038/s41598-021-98243-x

**Published:** 2021-09-27

**Authors:** Piyanoot Thongsri, Yongyut Pewkliang, Suparerk Borwornpinyo, Adisak Wongkajornsilp, Suradej Hongeng, Khanit Sa-ngiamsuntorn

**Affiliations:** 1 Department of Biochemistry, Faculty of Pharmacy, Mahidol University, Bangkok, 10400 Thailand; 2 Section for Translational Medicine, Faculty of Medicine Ramathibodi Hospital, Mahidol University, Bangkok, 10400 Thailand; 3 Excellent Center for Drug Discovery, Faculty of Science, Mahidol University, Bangkok, 10400 Thailand; 4 Department of Biotechnology, Faculty of Science, Mahidol University, Bangkok, 10400 Thailand; 5 Department of Pharmacology, Faculty of Medicine Siriraj Hospital, Mahidol University, Bangkok, 10700 Thailand; 6 Department of Pediatrics, Faculty of Medicine Ramathibodi Hospital, Mahidol University, Bangkok, 10400 Thailand

**Keywords:** Antivirals, Hepatitis B virus, Pharmacology, Target identification, Target validation

## Abstract

Hepatitis B virus (HBV) has been implicated in hepatitis and hepatocellular carcinoma. Current agents (nucleos(t)ide analogs and interferons) could only attenuate HBV infection. A combination of agents targeting different stages of viral life cycle (e.g., entry, replication, and cccDNA stability) was expected to eradicate the infection. Curcumin (CCM) was investigated for inhibitory action toward HBV attachment and internalization. Immortalized hepatocyte-like cells (imHCs), HepaRG and non-hepatic cells served as host cells for binding study with CCM. CCM decreased viral load, HBeAg, HBcAg (infectivity), intracellular HBV DNA, and cccDNA levels. The CCM-induced suppression of HBV entry was directly correlated with the density of sodium-taurocholate co-transporting polypeptide (NTCP), a known host receptor for HBV entry. The site of action of CCM was confirmed using TCA uptake assay. The affinity between CCM and NTCP was measured using isothermal titration calorimetry (ITC). These results demonstrated that CCM interrupted HBV entry and would therefore suppress HBV re-infection.

## Introduction

HBV could induce acute and chronic hepatitis that eventually led to liver cirrhosis and hepatocellular carcinoma (HCC)^1^. HBV belongs to the *Hepadnaviridae* family carrying a small envelope and partially double-stranded DNA. The HBV envelope consists of large (L), middle (M), and small (S) proteins encoded from a single open reading frame (ORF). The L-protein contains preS1 and preS2 sites that bind specifically to host receptors (i.e., NTCP and HSPGs)^2,3^. HBV and HDV entered host cells through endocytosis mediated by sodium-taurocholate co-transporting polypeptide (NTCP) and enhanced by heparan sulfate proteoglycans (HSPGs)^4–6^. Current HBV treatment includes Pegylated interferon (peg-IFN) and nucleos(t)ide analogs (NAs). NAs could not eradicate HBV but generated unbearable adverse effects^7–9^.

HBV entry involved two steps. HBV virion was initially attached to heparan sulfate proteoglycans (HSPGs) on the hepatocyte surface^5^ using the preS region of the L protein and the antigenic loop (Arg- or Lys-122 and Lys-141) of the viral S protein^10^. Subsequently, the virus shifted to its high-affinity receptor, NTCP. The pretreatment of host cells with heparin or cyclosporine A that targeted viral receptors could inhibit HBV infection. Other compounds (e.g., myrcludex B, irbesartan, ezetimibe, ritonavir, vanitaracin A, bile acids, etc.) that targeted NTCP reproduced the inhibition^11^.

Several agents targeting viral binding, entry, and replication have been developed. Blocking host receptor would rationally interfere viral entry and spreading^12,13^. The combination of HBV entry inhibitor and NAs was proposed to achieve a functional cure^14^. Only a few cultured cell models allowed HBV entry and replication. These included primary human hepatocyte (PHH) and differentiated HepaRG (d-HepaRG)^15,16^. PHH had limited life-span while d-HepaRG was barely infected by HBV due to its low NTCP level^17^. HepG2 transduced with ectopic NTCP (HepG2-hNTCP) was developed to screen HBV entry inhibitors^18,19^. All HepG2-derived cell lines had shortcomings of low CYP450s (i.e., CYP3A4, CYP2C9) and defective HBV life cycle^20,21^. The immortalized hepatocyte-like cell (imHC) was considered suitable owing to its native expression of NTCP and other hepatocyte markers, including. imHC could host the entire HBV life cycle^22,23^.

Herbal compounds (e.g., quercetin, epigallocatechin gallate (EGCG)^24,25^, silymarin^26^, naringenin^27^, including curcumin (CCM, Figure S1A)^28^) were investigated for anti-viral activities. Curcumin (diferuloylmethane) is a major phytochemical compound in the rhizome of *Curcuma longa* and members of *Curcuma* Spp.^29^. Curcuminoid derivatives consist of curcumin, demethoxycurcumin (DMC) and bisdemethoxycurcumin (BDMC)^30^. CCM contained anti-inflammatory, anti-microbial, anti-oxidant, anti-tumor and anti-viral actions^31–34^. CCM decreased HBV viral load by lessening histone acetylation of cccDNA in infected HepG2.2.15^35^. CCM could inhibit both entry and post-entry of hepatitis C virus, dengue virus, and influenza virus^36–39^. In this study, we investigated the inhibitory action of CCM toward NTCP-mediated HBV entry. imHCs and others were used as host cells for HBV infection^22^. Binding affinity was determined using isothermal titration calorimetry. CCM lessened HBV entry through NTCP binding. This resulted in decreasing all HBV viral makers in infected hepatocytes.

## Results

### Prophylactic CCM treatment decreased viral load, intracellular DNA, HBeAg, HBcAg and cccDNA

**Figure 1:**
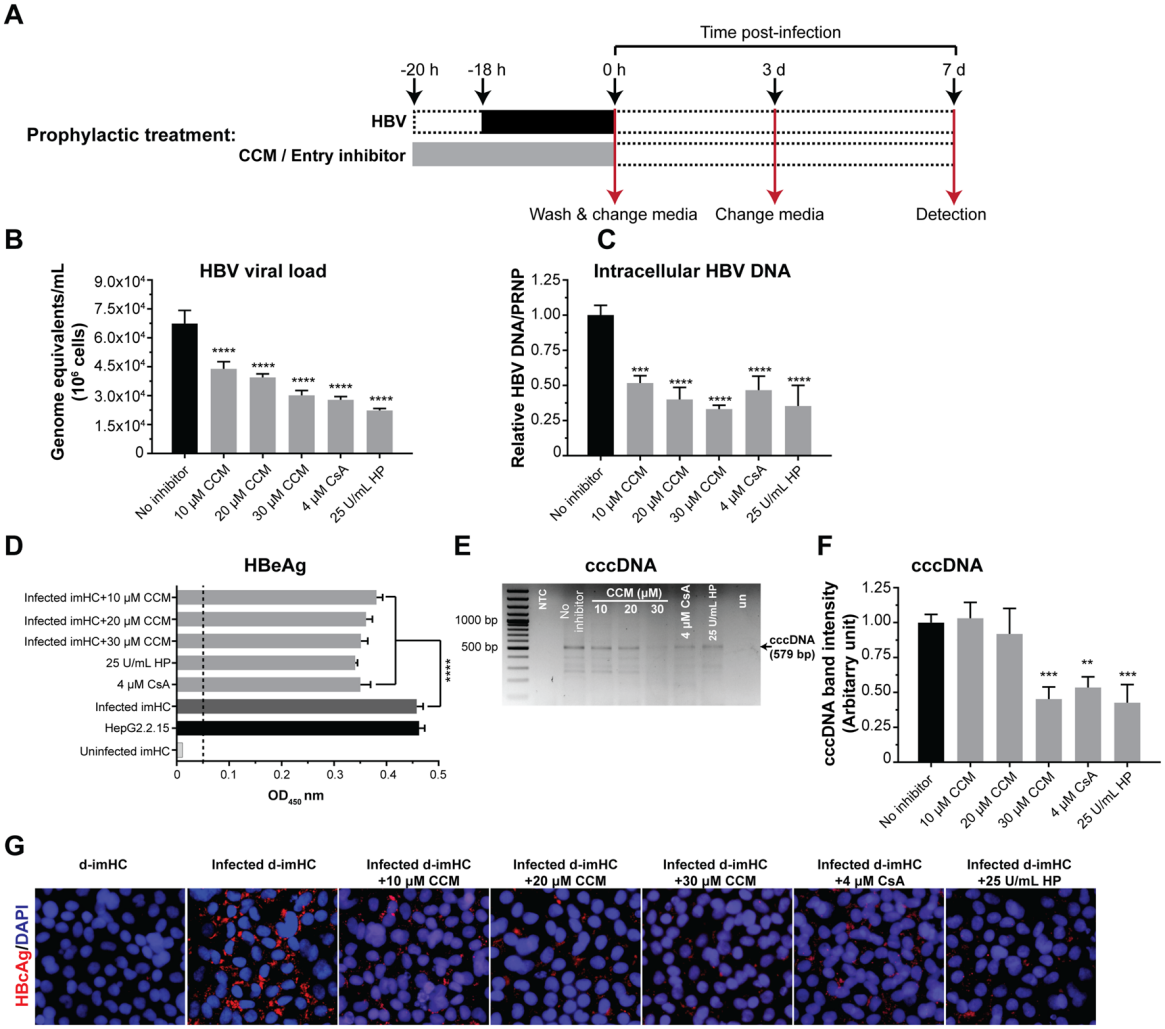
Prophylactic CCM treatment decreased viral entry, intracellular HBV DNA, viral load, HBeAg, HBcAg and cccDNA. d-imHCs were pretreated with CCM for 2 h prior to the inoculation with HBV **(A)**. On day 7 after infection, the decrease in extracellular HBV DNA (viral load) **(B)** as well as intracellular HBV DNA **(C)** by CCM was observed. CCM decreased secretory HBeAg in the conditioned medium **(D)**. The levels of HBV cccDNA was analyzed using agarose gel electrophoresis **(E)** and quantified **(F)**. Decreasing HBV infectivity (HBcAg) was observed through immunofluorescence **(G)**. Data were presented as mean ± SD. *, **, *** and **** represented statistical difference with a *p*-value < 0.05, < 0.01, < 0.001 and < 0.0001 respectively.

The prophylactic treatments (Fig. 1A) with 10, 20, 30 µM CCM, CsA, and HP decreased the viral load by 35%, 41%, 55%, 59% and 67% respectively (Fig. 1B). CCM up to 30 µM had no cytotoxic effect on imHCs (Figure S1B). CCM at 30 µM could lessen HBV viral load equivalent to that by 4 µM CsA (Fig. 1B). For intracellular HBV DNA, 10, 20, 30 µM CCM, CsA and HP decreased intracellular HBV DNA by 48%, 60%, 67%, 54% and 65% respectively (Fig. 1C). The level of HBeAg in the supernatant on day 7 post-infection could be suppressed with 10, 20 and 30 µM CCM in a dose-dependent manner (Fig. 1D). The HBV cccDNA level was lessened by a half after 30 µM CCM prophylactic treatment in infected-imHCs (Figs. 1E and 1F). CCM prophylactic treatment also decreased HBcAg in infected-imHCs based on the immunofluorescent staining (Fig. 1G).

### The suppression of HBV attachment and internalization by CCM depended on NTCP level

**Figure 2:**
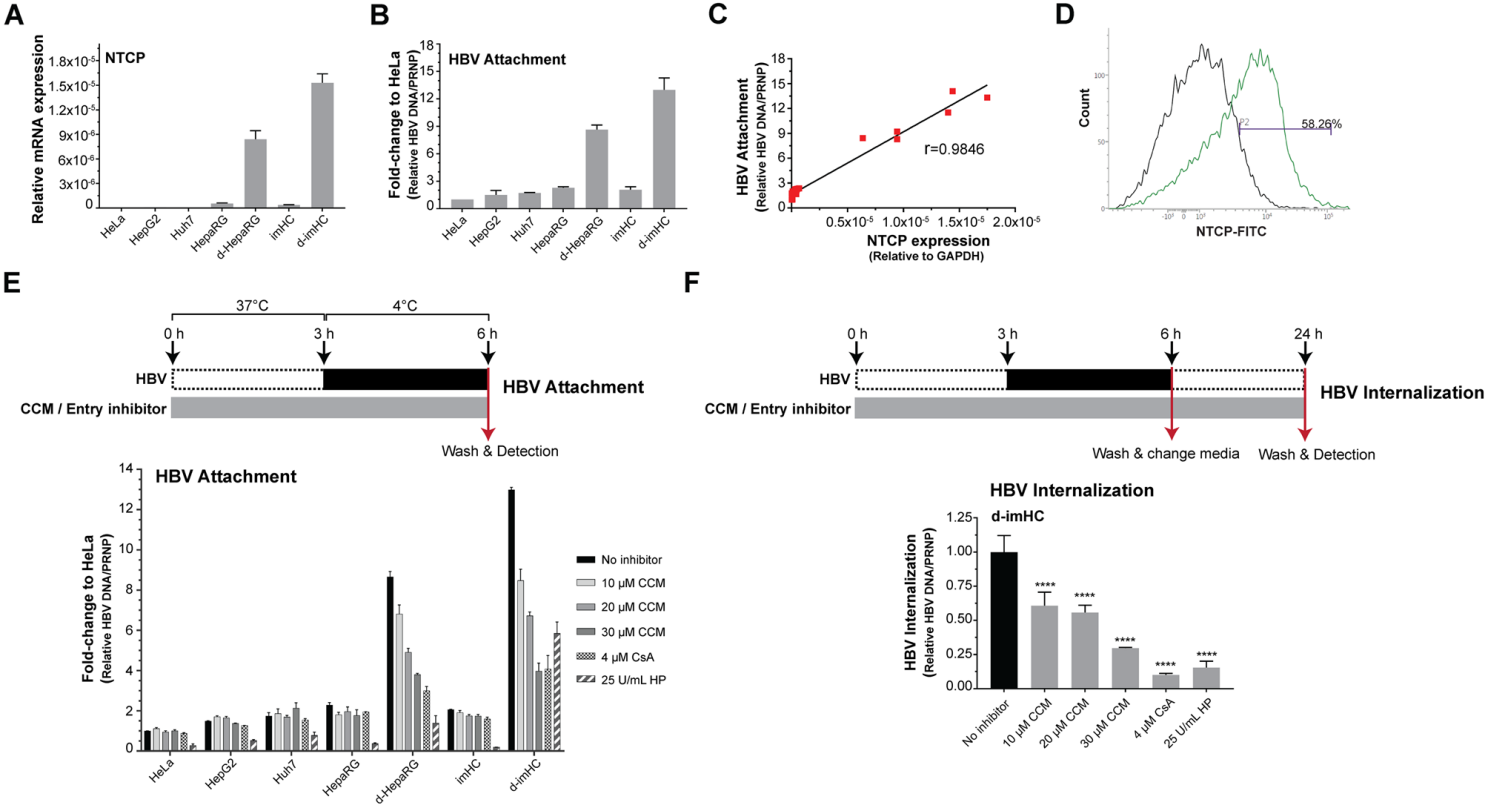
The inhibition of HBV attachment and internalization by CCM was depending on NTCP level. NTCP expression levels in HeLa, HepG2, Huh7, HepaRG, imHC, d-HepaRG and d-imHC was determined using qRT-PCR **(A)**. The levels of cell bound HBV DNA that represented immediate HBV attachment was measured using real-time qPCR **(B)**. A direct correlation between NTCP levels and the amount of bound HBV DNA was observed using Pearson's *r* **(C)**. NTCP density in d-imHC was evaluated by flow cytometry **(D)**. CCM inhibited HBV attachment in dose-dependent manner **(E)**. CsA and HP served as known entry inhibitors. CCM inhibited HBV internalization in d-imHC **(F)**. Data were presented as mean ± SD. *, **, *** and **** represented statistical difference with a *p*-value < 0.05, < 0.01, < 0.001 and < 0.0001 respectively.

The differentiation of HepaRG and imHC with DMSO, 5 μg/mL insulin and 50 μM hydrocortisone could upregulate the expression of NTCP. However, NTCP expression was undetectable and uninducible in HeLa, HepG2 and Huh7 (Fig. 2A). HBV inoculation with host cells for 3 h at 4 °C allowed HBV attachment, but not internalization (Fig. 2E). HBV attachment (bound HBV DNA) was increased by 4.5 and 6.5-folds in d-HepaRG and d-imHC respectively (Fig. 2B). The attachment directly correlated with NTCP levels on host cells (Fig. 2C). The differentiation of imHC increased NTCP by 58.26% (Fig. 2D). d-imHCs possessed the highest NTCP level as well as the highest HBV attachment (Fig. 2A,B). Prophylactic treatment of hepatocytes with CCM lessened HBV attachment. CCM at 10, 20 and 30 µM decreased the HBV DNA levels by 30%, 50% and 74% respectively in d-imHC; 20%, 40% and 55% respectively in d-HepaRG. HP and CsA pretreatment decreased the HBV attachment by 85% and 65% respectively. CCM could slightly decrease HBV uptake in undifferentiated imHCs and undifferentiated HepaRG that could be explained by their relatively lower NTCP expression than that of the differentiated cells (Fig. 2E). The schematic diagram to evaluate inhibitory effect of CCM on HBV internalization was illustrated (Fig. 2F). Prophylactic treatment of hepatocytes with CCM decreased intracellular HBV DNA (internalization). CCM at 10, 20 and 30 µM decreased HBV internalization by 39%, 44% and 73% respectively in d-imHC; but CsA and HP pretreatment decreased HBV internalization by 90% and 85% respectively (Fig. 2F). These suggested that CCM inhibited HBV attachment and internalization through NTCP receptor.

### CCM treatment at early phase and replication phase of HBV life cycle resulted in decreasing intracellular HBV DNA, viral load, and HBeAg

**Figure 3:**
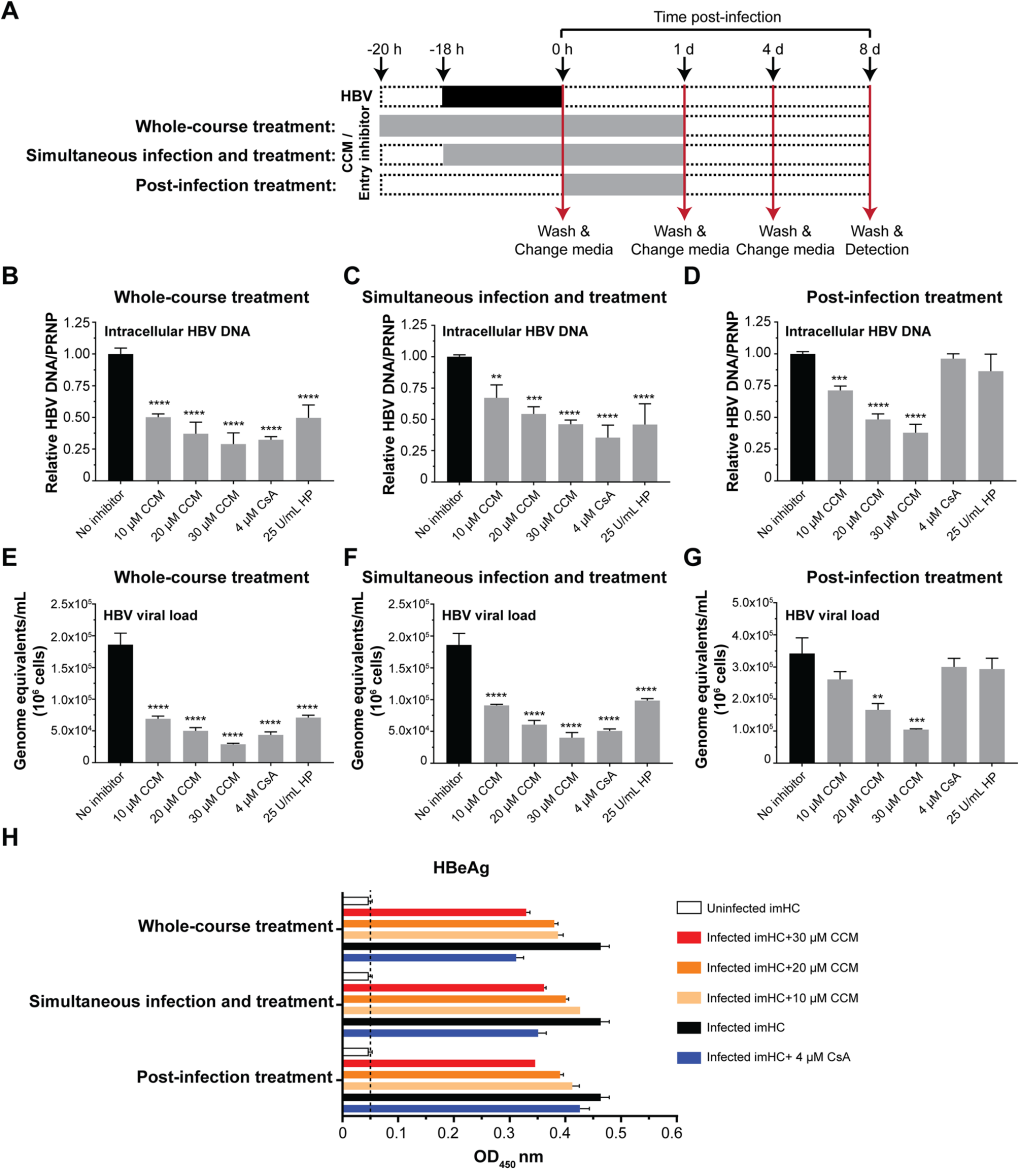
The combination of prophylactic treatment and post-viral entry with CCM enhanced anti-HBV activity. Schematic protocols represented treatment strategies with 10, 20 and 30 µM CCM **(A)**. The reduction of intracellular HBV DNA in whole-course treatment **(B)**, simultaneous infection and treatment **(C)** and post-infection treatment **(D)** with 10, 20 and 30 µM CCM significantly decreased intracellular HBV on day 8 after infection. The HBV viral load was decreased in whole-course treatment **(E)**, simultaneous infection and treatment **(F)** and post-infection treatment **(G)** with CCM. CCM also decreased HBeAg in infected-imHC conditioned medium **(H)**. Data were presented as mean ± SD. *, **, *** and **** represented statistical difference with a *p*-value < 0.05, < 0.01, < 0.001 and < 0.0001, respectively.

Several studies reported that CCM acted over HBV replication phase (transcription and nucleocapsid assembly). However, the effect of CCM on early phase (attachment through NTCP and/or internalization) has yet been investigated. The anti-viral activity of CCM in whole-course treatment (early and replication phases), simultaneous infection treatment and post-infection treatment were evaluated (Fig. 3A). On day 8, CCM at 10, 20 and 30 µM decreased intracellular HBV by 50%, 63% and 71% respectively, while CsA and HP decreased the intracellular HBV by 67% and 50% respectively (Fig. 3B). In simultaneous infection and treatment, 10, 20 and 30 µM CCM decreased intracellular HBV by 33%, 46% and 54% respectively, while CsA and HP decreased the HBV viral load by 64% and 54% respectively (Fig. 3C). CCM decreased intracellular HBV in post-infection treatment in the same fashion as that in simultaneous infection and treatment, but CsA and HP did not alter intracellular HBV DNA (Fig. 3D). For viral production, whole-course treatment with 10–30 µM CCM decreased HBV viral load by 2.69–6.43 folds (Fig. 3E). The simultaneous infection and treatment decreased viral load in the same fashion as that in whole-course treatment (Fig. 3F). For post-infection treatment, 20–30 µM CCM decreased HBV viral load but CsA and HP no longer had detectable effect (Fig. 3G). CCM also decreased the HBeAg level in the imHC conditioned medium in a dose-dependent manner. The inhibitory effect of CsA in each treatment condition served as a control (Fig. 3H). Therefore, CCM carried anti-HBV activity in the whole-course treatment and the post-infection treatment. CCM could inhibit HBV propagation in the early phase as well as the replication phase. The combination of CCM and entecavir (ETV) exhibited synergistic inhibition of HBV replication in post-infection treatment (Figure S2C) and whole-course treatment (Figure S2D). This synergistic action suggested CCM and ETV acted through different mechanisms.

### The CCM uptake was dependent on Na^+^ concentration and competed with TCA

**Figure 4:**
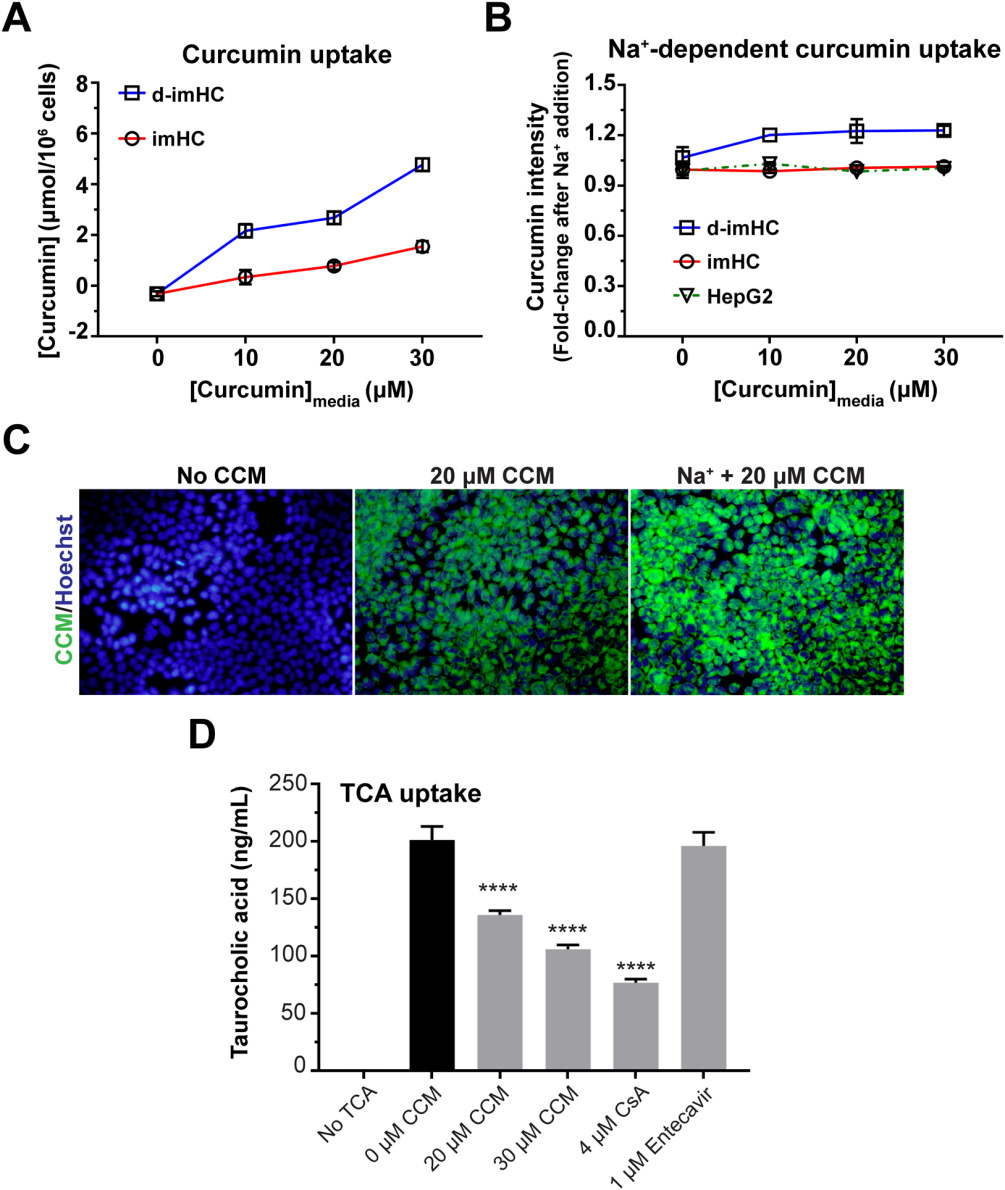
CCM was taken up by imHC and d-imHC and exerted suppressive effect on cellular TCA uptake. To investigate whether cellular uptake of CCM was in dose-dependent manner, cells were treated with 0–30 µM CCM for 16 h and sonicated into cell lysate. Cellular level of CCM was analyzed in cell lysate through a fluorescent microplate reader and quantitated using a standard curve with R^2^ = 0.9907 **(A)**. The effect of Na^+^ addition on CCM uptake was measured and presented as fold-changes over that with regular medium **(B)**. The representative fluorescent images of d-imHC treated with 20 µM CCM, ± Na^+^ addition were qualitatively visualized **(C)**. The inhibitory effect of CCM on TCA uptake was measured **(D)**. Data were presented as mean ± SD. *, **, *** and **** represented statistical difference with a *p*-value < 0.05, < 0.01, < 0.001 and < 0.0001, respectively.

To investigate whether CCM was taken up through NTCP, d-imHC and imHC were incubated with 0—30 µM CCM. The incubation with 30 µM CCM for 5 min–16 h, the intracellular CCM was increased from 0 to 1–2 µmol/10^6^ cells in imHC and 4–6 µmol/10^6^ cells in d-imHC (Figure S3A). The intracellular CCM level in d-imHC was higher than that of imHC in a dose-dependent manner (Fig. 4A and S3B). NTCP transport function could be driven by Na^+^ gradient. Increasing Na^+^ concentration in culture medium magnified CCM uptake into d-imHC by 1.2 folds (Fig. 4B) as observed under fluorescence microscope (Fig. 4C). To clarify whether CCM could interrupt NTCP-mediated taurocholic acid (TCA) uptake, d-imHC was treated by 0–30 µM CCM for 2 h prior to the incubation with TCA for 15 min. Interestingly, 20 and 30 µM CCM pretreatment lessened TCA uptake by 23.61% and 47.43% respectively, while CsA lessened TCA uptake by 61% (Fig. 4D). The combination of CCM with CsA could not reach synergistic response in the suppression of TCA uptake (Figure S2E). This suggested CCM shared the same mechanism as did CsA.

### CCM inhibited HBV infection through NTCP binding

**Figure 5:**
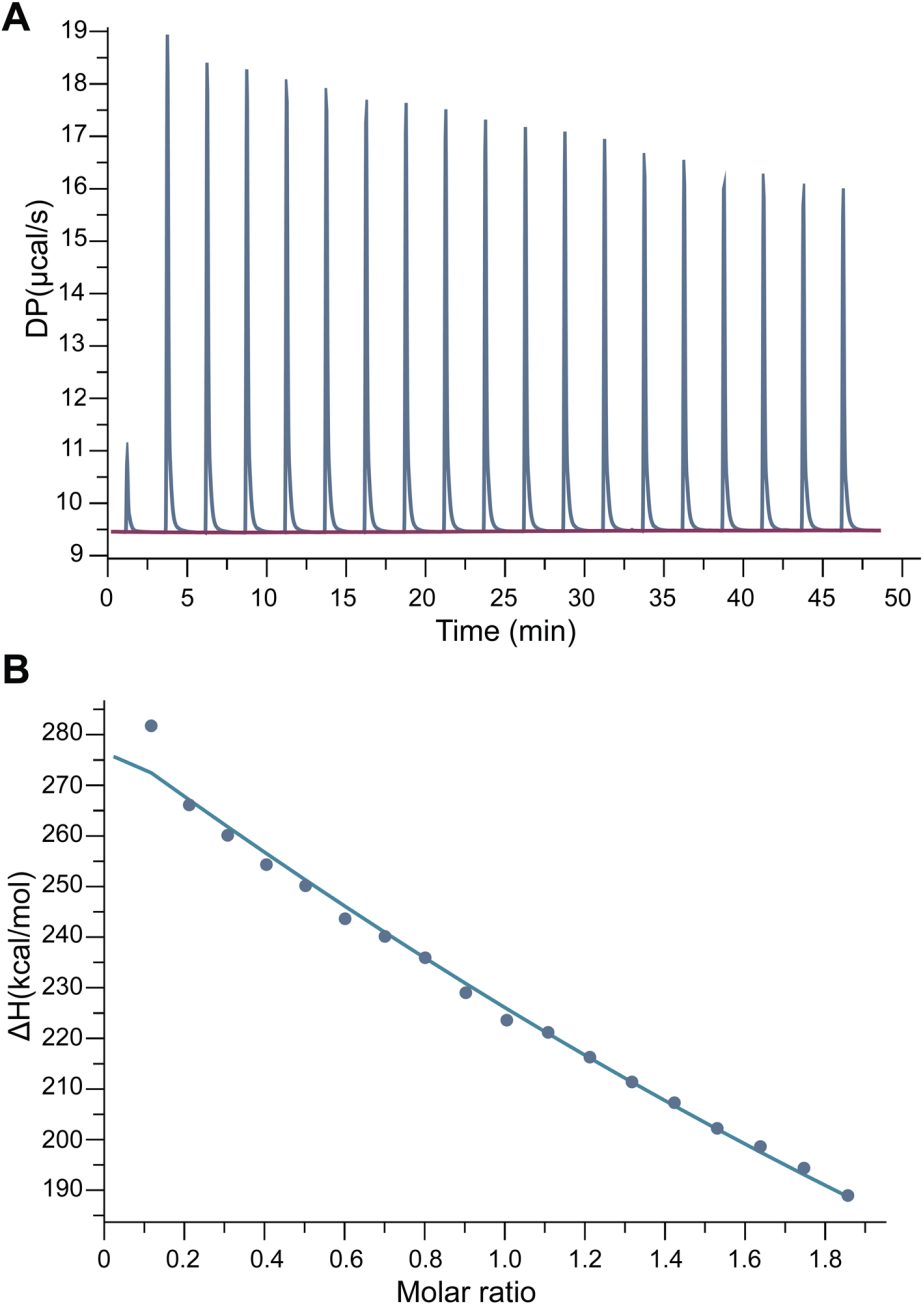
Isothermal titration calorimetric (ITC) profile of the binding between CCM and NTCP. The titration of raw data between differential power (DP, µcal/s) and time (min) after sequential injection of ligand (150 μM CCM) into the protein solution (15 μM of NTCP, pH 7.0, 25 °C) was plotted **(A)**. The integrated heat after the correlation between enthalpy changes versus the molar ratios of CCM (injection) to protein (NTCP) was plotted **(B)**. Data were fitted using a one-site binding model where the solid lines represented the best-fit results.

The thermodynamic parameters for the association between 2 molecules were determined using isothermal titration calorimetry (ITC). These parameters included the stoichiometry (n), the dissociation constant (K_D_), the change in free energy (ΔG), enthalpy (ΔH), entropy (ΔS), and heat capacity of binding (ΔCp). ITC operated by measuring heat alteration after the association of a ligand with its binding partner. To elucidate whether CCM inhibited HBV entry through NTCP binding, the specificity of the interaction between NTCP and CCM had to be assessed. This interaction might interfere the binding of HBV to NTCP. A representative calorimetric titration profile of human NTCP was elucidated (Fig. 5A). Data represented the titration of 15 μM NTCP with 150 µM CCM at pH 7.6, 25 °C. The calorimetric response was measured as successive injections were added to the sample cell. A standard nonlinear least squares regression binding model involving one binding site fitted well to the data. The solid line was the best fit to the experimental values (Fig. 5B and Figure S4). Thermodynamic data associated with the binding of NTCP and CCM were summarized (Table S1). The energy characteristic was endothermic as evidenced by positive enthalpy (ΔH) at 80.0 kcal/mol and free energy change (ΔG) at -5.72 kcal/mol. The data indicated that the binding was stable (Table S1). CCM could bind NTCP with an equilibrium dissociation constant (K_D_) of 6.43 × 10^–5^ M comparing to 2.56 × 10^–10^ M for TCA (natural ligand) and 9.29 × 10^–6^ M for CsA (Table S1). This suggested that CCM and NTCP have mutual hydrophobic interaction. When HBsAg was selected as target protein, CCM displayed no binding activity toward HBsAg (Figure S5A,B). Tris–HCl loading buffer and HBsAg ligands served as negative controls (Figure S5C,D).

## Discussion

HBV entry started through the low affinity binding to host heparan sulfate proteoglycans (HSPGs)^5,40^ followed by the binding to NTCP and the ensuing NTCP-receptor mediated endocytosis^41^. Inhibition of viral entry would be clinically translated to blocking de novo infection, vertical transmission, and recurrence after liver transplantation. Interfering viral entry could be a feasible alternative treatment for chronic hepatitis B. Myrcludex-B, an HBV entry inhibitor, could suppress HBV viral load in active infection^10,42,43^. However, myrcludex-B was not orally bioavailable and was immunogenic owing to its nature as an acidic peptide. Orally administered nonimmunogenic natural products possessing viral entry suppression would offer a better alternative to myrcludex-B.

CCM could suppress the infectivity of diverse enveloped viruses including influenza, dengue virus type II, Japanese encephalitis virus (JEV), pseudorabies virus (PRV), zika and chikungunya virus^28,44^. It interfered HCV binding and fusion^36^. CCM inhibited cccDNA-bound histone acetylation and decreased HBV viral load^35^. The precise antiviral activities of CCM had not been clearly elucidated.

NTCP was exclusively expressed in hepatocytes in animal models such as chimpanzees and tree shrews (*Tupaia belangeri*)^45^. Earlier studies developed NTCP-expressing HepG2 that allowed HBV entry and infection^46^. Since NTCP-expressing hepatocytes were established, the precise mechanism in early stages of HBV and HDV infections was revealed^22,45,46^. d-imHC, with natively high NTCP level, could effectively host HBV infection, propagation and spreading^22^. imHC functioned as a fitting hepatocyte alternative as it served as an effective host for human malarial parasite and Dengue virus infection^47,48^.

We proposed CCM as a candidate for inhibiting HBV infection using d-imHC as a host. Firstly, we determined CCM concentrations that elicited antiviral activity. CCM at 10–30 µM decreased intracellular HBV DNA by 67% and HBV viral load by 55% without hepatotoxicity similar to those treated with either CsA or HP^49^. CCM also lessened HBeAg, HBcAg and cccDNA. Secondly, the antiviral activity was investigated whether it involved the blocking of viral entry via NTCP. The association between NTCP level, CCM and HBV infectivity were studied in several cell lines. These included NTCP-expressed cells (d-imHC, d-HepaRG) and non-NTCP-expressed cells (HepG2, Huh7, and HeLa). A direct correlation between NTCP levels and the HBV attachment/internalization was observed. Only NTCP-expressing hepatocytes exhibited lessening intracellular HBV DNA after the CCM treatment. These suggested that the decrease in intracellular HBV particles after CCM treatment could be mediated through NTCP. Intracellular HBV particles were lessened in the same fashion after the pretreatment with classical HBV entry inhibitors (i.e., HP and CsA)^49^. CsA is a cyclic peptide that binds NTCP while HP binds heparan sulfate proteoglycans (HSPGs)^4^.

NTCP acted as a Na^+^-dependent co-transporter to uptake bile acids from plasma for enterohepatic circulation of bile acids^11^. NTCP also served as a receptor for receptor-mediated endocytosis during viral entry^40^. We found that d-imHC, but not undifferentiated imHC nor HepG2, was rich in NTCP and highly accumulated CCM. In addition, the addition of Na^+^ enhanced the CCM uptake in d-imHC, but not in imHC nor HepG2, which implied the role of NTCP in CCM uptake. CCM also inhibited NTCP function which should otherwise mediate TCA uptake in d-imHC. These observations indicated that CCM inhibited HBV attachment and internalization through NTCP.

The anti-HBV activities of CCM were reported. Most of the publications concluded that CCM inhibited HBV infection at post-viral entry (transcription, nucleocapsid assembly)^35,50,51^. However, these studies used HepG2.2.15 that could not reliably assess the anti-HBV activity at the early phase of HBV lifecycle (attachment/entry/nuclear trafficking/and cccDNA formation). The anti-HBV activity of CCM in 4 settings (prophylactic treatment, simultaneous infection and treatment, post-infection treatment and whole-course treatment) was investigated. We found that the combination of prophylactic treatment and post-infection treatment optimally suppressed the HBV viral load and intracellular HBV DNA as oppose to either prophylactic treatment or post-infection treatment alone. These findings indicated that CCM could interrupt HBV infection in various stages of HBV life cycle.

ITC was frequently used to analyze the prospect of interactive binding that would identify inhibitors and their target proteins. The binding affinity (K_D_) of biomolecules through non-covalent interactions of the molecule complex formation could be obtained^52,53^. ITC was applied to identify the protease inhibitor of HIV that could inhibit viral replication^54,55^. We used ITC to evaluate the binding affinity between CCM and a recombinant NTCP. CCM could spontaneously bind NTCP as indicated by −ΔG (−5.72 kcal/mol) and endothermic (+ ΔH) reaction. Several herbal anti-HBV agents had been analyzed by molecular docking (e.g., quercetin, rutin, hesperidin, luperol, azadirachtin, beta-sitosterol, psoralen, embelin, menisdaurin and baccatin III) could specifically bind HBV reversed transcriptase comparable to lamivudine, a nucleoside analog. These compounds exhibited negative Gibb energy (−ΔG) ranging from −9.3 to −5.2 kcal/mol and K_D_ of 10^–6^ to 10^–3^ M with HBV polymerase^56^.

Although CCM was proposed as a HBV entry inhibitor, the poor aqueous solubility and relatively low bioavailability posted a limitation for clinical application^57^. Several CCM formulations were designed to overcome this limitation^58^. The free CCM concentrations in human plasma after taking 4, 6 and 8 g CCM daily for 3 months were 0.52, 0.54, and 1.63 µM respectively^59^. The total CCM glucuronides and sulfates conjugated in six healthy volunteers after taking 10 g of CCM was 8.42 µM^60^. Our study used CCM at working concentrations ranging from 10 to 30 µM, which were higher than those observed in human plasma at the given long-term doses. Interestingly, in Wistar rat, 227.5 µM, 1.33 mM, and 65 µM CCM were found in serum, whole blood, and liver respectively at 6 h after a single oral administration of 500 mg/kg^61^ without any serious adverse effect. Based on these data, it is possible to reach 30 µM free CCM in human plasma through adjusted CCM doses and formulations. Taken together, CCM acted as an HBV entry inhibitor through the binding with NTCP, the first step of HBV infection.

## Materials and methods

### Cell culture

imHC was maintained in 1:1 DMEM/F12 (Hyclone). imHC could harbor the entire HBV life cycle^22^. HepaRG (Thermo Fisher Scientific, Waltham, MA, USA), Huh7 and HepG2 (ATCC, Manassas, VA, USA) were maintained in DMEM/F12 (Hyclone). HeLa cell was maintained in DMEM (Hyclone). All media were supplemented with 10% FBS (Hyclone), 100 U/mL penicillin and 100 µg/mL streptomycin (Invitrogen, USA) at 37 °C with 5% CO_2_. Prior to HBV infection, imHCs and HepaRG were differentiated with 2% DMSO (Sigma, NY) in Williams’ E medium, 10% FBS, 100 U/mL penicillin, 100 μg/mL streptomycin, 5 μg/mL insulin, 50 μM hydrocortisone, 2 mM l-glutamine (GlutaMax, Gibco, Thermo Fisher Scientific, MA) for 2 weeks.

### Production of the cell culture-derived HBV particles (HBVcc)

The stable HBV transduced HepG2, clone 2.2.15, was used to generate HBV particles. HepG2.2.15 was maintained in DMEM, 10% FBS, 100 IU/ml penicillin and 100 µg/ml streptomycin (Invitrogen, USA), and 380 µg/mL G418 (geneticin). The conditioned medium was harvested for viral particles every 7 days. The viral particles were 100 × concentrated with Lenti-X concentrator (Clonetech, USA) after filtered through 0.45 μm. The 100 × of HBV was quantified, aliquoted and stored at − 80 °C until use.

### Cytotoxic assay of CCM in hepatocytes

CCM (C1386, Sigma, St. Louis, MO) was dissolved in absolute ethanol (EtOH) at 5 mg/mL and stored at − 40 °C. The cytotoxicity of CCM on imHC was monitored using MTT assay^62^. Briefly, imHCs were seeded on 96-well plate at 2 × 10^5^ cells/mL overnight at 37 °C, 5% CO_2_. The imHC at 90% confluency was treated with 0–50 µM CCM for 24 h. Cell viability was assessed using MTT assay with a microplate reader (Tecan, Switzerland) at 570 nm absorbance.

### The inhibitory effect of CCM on HBV attachment and internalization

HepG2, Huh7, HepaRG, imHC and HeLa cells contained different expression levels of NTCP. For HBV attachment assay, cells were treated with an entry inhibitor for 3 h before the inoculation with HBV for 3 h at 4 °C to prevent HBV internalization. CsA and HP were positive controls for blocking HBV entry through NTCP and heparan sulfate proteoglycans (HSPGs) respectively. Infected cells were vigorously washed thrice with cold PBS to remove non-specifically bound viral particles. For HBV internalization assay, d-imHC was pretreated with CCM or an entry inhibitor for 3 h before the inoculation with HBV for 3 h at 37 °C for additional 3 h. Infected cells were washed thrice and cultured for 24 h in complete medium supplemented with CCM or an entry inhibitor. The cell was trypsinized to remove plasma membrane-bound HBV particles. Cell pellet was harvested to isolate intracellular HBV DNA.

### The uptake of CCM and TCA in imHCs

imHC and d-imHC were incubated with 0, 10, 20 or 30 µM of CCM up to 16 h. After incubation, cells were vigorously washed thrice and harvested by trypsinization. Cell pellets were suspended in methanol and lysed using ultrasonication to dissolve CCM into methanol fraction. Cell debris was separated from methanol fraction through centrifugation. The dissolved CCM was measured by fluorescence colorimetric method with 435 nm excitation and 535 nm emission using Clariostar Microplate Reader. CCM concentration was extrapolated with a standard curve of CCM standard^63^. The effect of Na^+^ level on CCM uptake via NTCP was investigated. A range of 152.30–165 mM Na^+^ in culture medium was added together with 20 µM CCM for 16 h. CCM uptake was measured and presented as fold-changes after Na^+^ addition. Taurocholic acid (TCA) uptake activity was monitored in imHC and d-imHC. Cells were treated with CCM or an entry inhibitor for 2 h at 37 °C followed by the addition of taurocholic acid for 15 min. Cellular TCA uptake was measured with a liquid scintillator.

### The anti-HBV activity of CCM under different exposure conditions

To determine whether the anti-viral activity of CCM took place over either the early phase or the replication phase, four treatment conditions were formulated. These consisted of (1) prophylactic treatment, (2) simultaneous infection and treatment, (3) post-infection treatment, and (4) whole-course treatment. For prophylactic treatment, differentiated imHCs (d-imHCs) were preincubated with 0, 10, 20, 30 µM CCM or an HBV entry inhibitor (4 µM cyclosporine A, CsA; or 25 units/mL heparin, HP, Sigma, MO) for 2 h, inoculated with HepG2.2.15-derived HBV particles (HBVcc) at MOI 100 in Williams’ Media E, 4% polyethylene glycol (PEG) for 18 h, washed and maintained in complete medium (Williams’ medium E, 10% FBS, 100 IU/ml penicillin and 100 µg/ml streptomycin) for 7 days. For simultaneous infection and treatment, d-imHCs were inoculated with HBV together with CCM or entry inhibitors for 18 h, washed, incubated with the same chemical for 24 h, washed and maintained in complete medium for 7 days. For post-infection treatment, d-imHCs were inoculated with HBVcc for 18 h, washed, treated with the same chemical for 24 h, washed and maintained in complete medium for 7 days. For whole-course treatment, d-imHCs were treated with CCM or entry inhibitor for 2 h, inoculated with HBVcc for 18 h, washed, incubated with the same chemical for 24 h, washed and maintained in complete medium for 7 days. The conditioned media on the last day were collected and determined for viral load and HBeAg level. Cell pellets on the last day were collected for the quantitation of HBV cccDNA and intracellular HBV DNA using real-time qPCR. The primary outcomes consisted of viral load, intracellular DNA, HBeAg and cccDNA.

### The detection of HBV DNA, cccDNA and NTCP expression by quantitative real-time PCR

The harvested cells and the conditioned media containing HBV were extracted for total DNA or HBV DNA using the Nucleospin kit (MN, Düren, Germany). Quantitative real-time PCR was performed by mixing 50 ng total DNA or 2 µL of HBV DNA with KAPA SYBR FAST qRT-PCR Kit solution (Kapa Biosystems, USA). The PCR master mixture was amplified using the Mx3000P qPCR System (Agilent Technologies, USA) with specific HBV primers for HBV DNA: the forward primer is 5’-GTTGCCCGTTTGTCCTCTAATTC-3’ and the reverse primer is 5’-GGAGGGATACATAGAGGTTCCTTGA-3’. PRNP was used as a calibrator gene. The PRNP forward primer is 5’-GACCAATTTATGCCTACAGC-3’; the reverse primer is 5’-TTTATGCCTACAGCCTCCTA-3’. The HBV cccDNA forward primer is 5’-GACTCTCTCGTCCCCTTCTC-3’; the reverse primer is 5’-ATGGTGAGGTGAACAATGCT-3’. The HBV DNA was quantified using plasmid HBV 1.3-mer WT replicon (Addgene plasmid # 65459) as a standard curve. The NTCP expression level in host cells was measured. The cell pellet was extracted for total RNA using GE Healthcare illustra™ RNAspin Mini Isolation Kit (GE Healthcare, USA). Total RNA was immediately converted to cDNA using ImProm-II™ Reverse Transcription System (Promega, WI). NTCP was amplified from 50 ng of cDNA using NTCP specific primers: 5’-GGACATGAACCTCAGCATTGTG-3’ and 5’-ATCATAGATCCCCCTGGAGTAGAT-3’. GAPDH was used as a house-keeping gene and amplified with the forward primer, 5’-GAAATCCCATCACCATCTTCC-3’, and the reverse primer, 5’-AAATGAGCCCCAGCCTTCTC-3’. To detect cccDNA in infected imHC, relaxed circular DNA (rcDNA) was excluded from the isolated HBV DNA using the NucleoSpin plasmid DNA extraction kit (MN, Düren, Germany). The total DNA was incubated with exonuclease to digest contaminating non-cccDNA forms. The cccDNA was amplified by specific primers: 5′-GACTCTCTCGTCCCCTTCTC-3′, and the reverse primer, 5′-ATGGTGAGGTGAACAATGCT-3′ with a PCR product of 580-bp fragment, which spanned the gap and the nick in the rc form of the HBV genome. The optimized PCR condition consisted of 95 °C for 5 s, 45 cycles at 95 °C for 15 s, 60 °C for 4 s, 72 °C for 25 s and detection at 88 °C for 2 s after each cycle. The specificity to amplify cccDNA over rcDNA appeared to be 10^4^ to 1. The authenticity of cccDNA amplification was confirmed by gel electrophoresis.

### The quantification of HBeAg using sandwich enzyme-linked immunosorbent assay

The amount of secreted e antigen (HBeAg) in the conditioned medium was evaluated using ELISA detection kit (KA0290, Abnova, Taiwan) following the manufacturer’s instruction. Colorimetric alternation was determined at 450 nm absorbance by Clariostar Plus Microplate Reader (BMG Labtech, Germany).

### Indirect immune fluorescence

The d-imHCs were pretreated with 10–30 µM CCM, inoculated with HBVcc at MOI 100 in Williams’ Media E, 4% PEG for 18 h, washed and maintained in complete medium for 7 days, fixed with 4% (w/v) paraformaldehyde and permeabilized with 0.2% (v/v) Triton-X100 in PBS, incubated with anti-HBcAg antibody (ab8637, Abcam, UK) at 4 °C overnight, incubated with Alexa Fluor 568-conjugated goat-anti-mouse IgG (A11004, Life Technologies, USA) for 1 h, counterstained with DAPI (Thermo Fisher Scientific, USA), and visualized under Nikon Eclipse E800 microscopy (Nikon, Japan).

### Thermodynamics of ligand–protein interaction

The molecular binding interactions between NTCP (SLA10A1) and CCM was analyzed by isothermal titration calorimeter (ITC) to determine the binding affinity (K_D_), stoichiometry (n), enthalpy (ΔH) and the molar Gibbs free energy (ΔG) at 25 °C. To examine NTCP and CCM interactions, 15 µM of a recombinant NTCP/SLC10A1 (Cloud-clone, USA) were reconstituted in 50 mM tris–HCl pH 7.6 and loaded into the sample cell. The tris–HCl buffer was added to the reference cell. CCM concentration was fixed at 150 µM. During the procedure, 2-µL aliquot of CCM solution was successively injected into a NTCP-containing cell. The titration curves alteration (ΔP/ΔT) was plotted and analyzed by Microcal PEAQ-ITC software (Malvern Panalytical UK).

### Statistical analysis

All experiments were performed in triplicate. Data were expressed as means ± SD, and the statistical analyses were performed using GraphPad Prism 7 software. For comparison between two mean values, a two-tailed unpaired students’ t test was used to calculate statistical significance. One-way Analysis of Variance (ANOVA) was used with Dunnett for multiple comparisons to compare multiple values to a single value, or with Tukey’s HSD to compare multiple values to each other. A *p*-value less than 0.05 was considered statistically significant.

## Supplementary Information

Supplementary Information.
