# Supplementary material for: Curcumin inhibited hepatitis B viral entry through NTCP binding
Source: Sci Rep. 2021 Sep 27;11:19125. doi: 10.1038/s41598-021-98243-x (PMC8476618; doi:10.1038/s41598-021-98243-x)
Supplement: Supplementary file 1 — Supplementary Information. [file 41598_2021_98243_MOESM1_ESM.docx]

**Curcumin inhibited hepatitis B viral entry through NTCP binding**

Piyanoot Thongsri P^1,2^P, Yongyut Pewkliang P^1,2^P, Suparerk Borwornpinyo P^3,4^P, Adisak Wongkajornsilp P^5,*^P, Suradej Hongeng P^6,*^P and Khanit Sa-ngiamsuntorn P^1,*^

P^1^PDepartment of Biochemistry, Faculty of Pharmacy, Mahidol University, Bangkok 10400, Thailand; piyanoot20@hotmail.co.th (P.T.); yongyut.pew@gmail.com (Y.P.)

P^2^PSection for Translational Medicine, Faculty of Medicine Ramathibodi Hospital, Mahidol University, Bangkok 10400, Thailand; piyanoot20@hotmail.co.th (P.T.); yongyut.pew@gmail.com (Y.P.)

P^3^PExcellent Center for Drug Discovery, Faculty of Science, Mahidol University, Bangkok 10400, Thailand; bsuparerk@gmail.com (S.B.)

P^4^PDepartment of Biotechnology, Faculty of Science, Mahidol University, Bangkok 10400, Thailand; bsuparerk@gmail.com (S.B.)

P^5^PDepartment of Pharmacology, Faculty of Medicine Siriraj Hospital, Mahidol University, Bangkok 10700, Thailand; adisak.won@mahidol.ac.th (A.W.)

P^6^PDepartment of Pediatrics, Faculty of Medicine Ramathibodi Hospital, Mahidol University, Bangkok 10400, Thailand; suradej.hon@mahidol.ac.th (S.H.)

*****Correspondence: khanit.san@mahidol.ac.th, K.S.; suradej.hon@mahidol.ac.th, S.H. and adisak.won@mahidol.ac.th, A.W.

**Supplementary Materials**


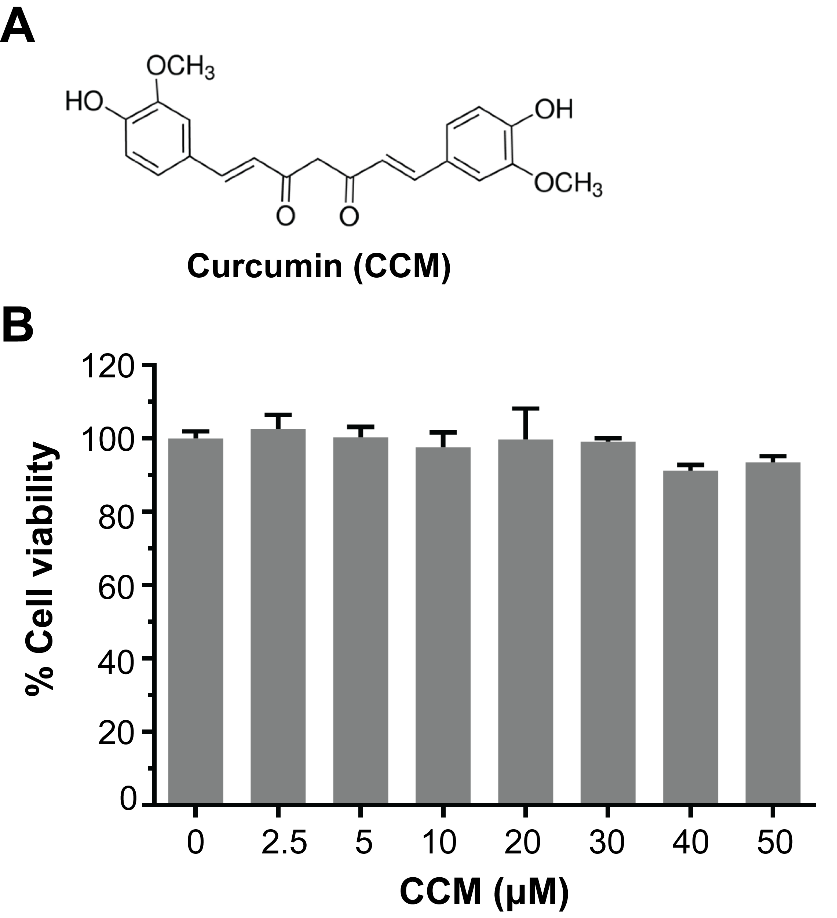


**Figure S1** The sublethal CCM concentration toward immortalized hepatocyte-like cell (imHC) was determined. After incubating CCM (A) up to 50 µM for 24 h. imHC displayed no cytotoxicity as monitored by MTT assay (B). Data were presented as mean ± SD.


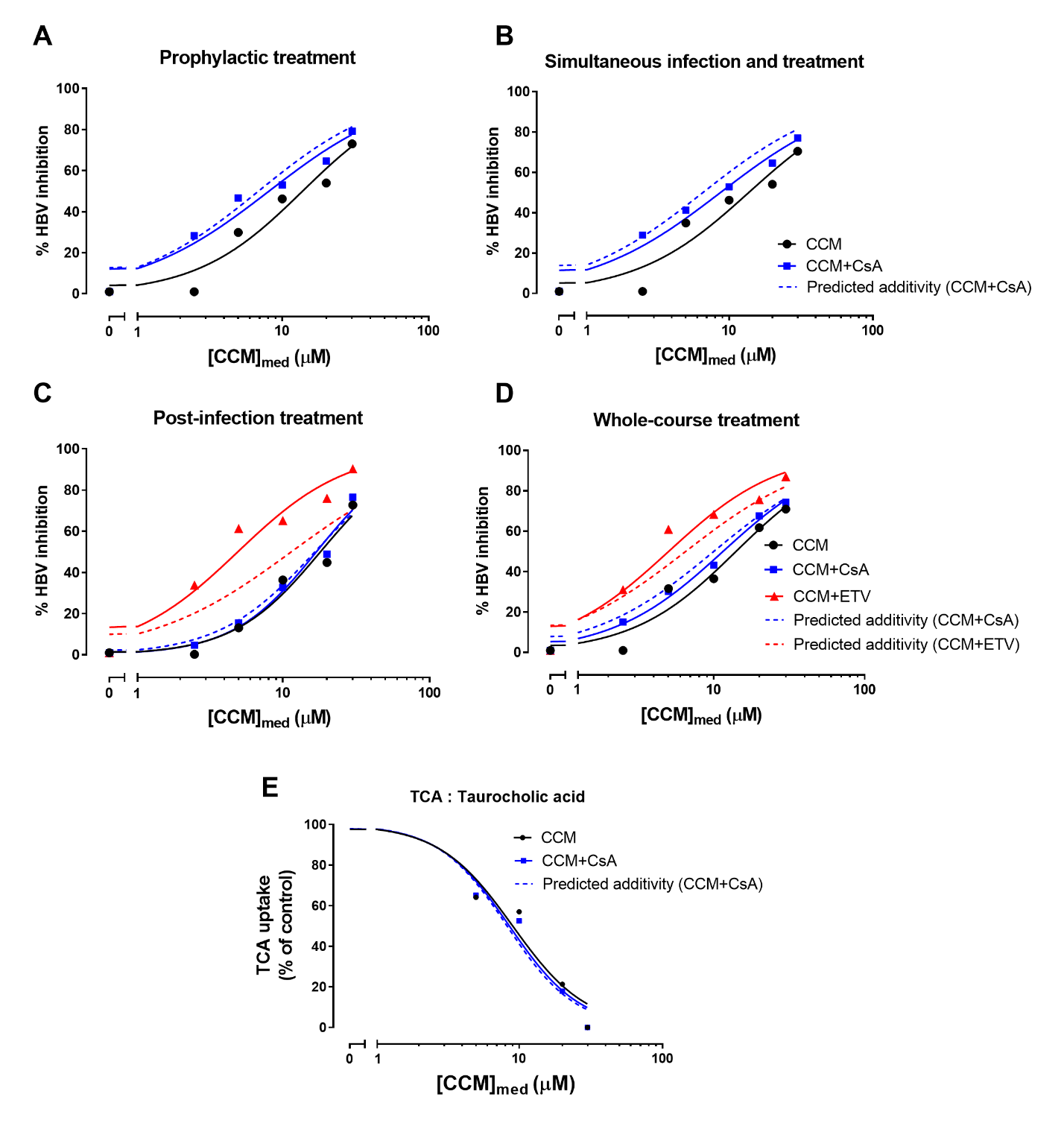


**Figure S2** CCM exhibited synergistic effect with entecavir (ETV) for the suppression of HBV replication in imHC. Bliss independence model was used to investigate the combination of CCM with either CsA or ETV toward the suppression of HBV replication. The additive effect of CCM plus CsA on the suppression of HBV DNA level was observed in prophylactic treatment (A) and simultaneous infection and treatment (B). CCM plus ETV exhibited synergistic effect on the suppressing HBV replication in post-infection treatment (C) and whole-course treatment (D). The combination of CCM with CsA barely exhibited additive response in the suppression of TCA uptake in imHC (E). The synergistic threshold lines were extrapolated using Bliss independence model.


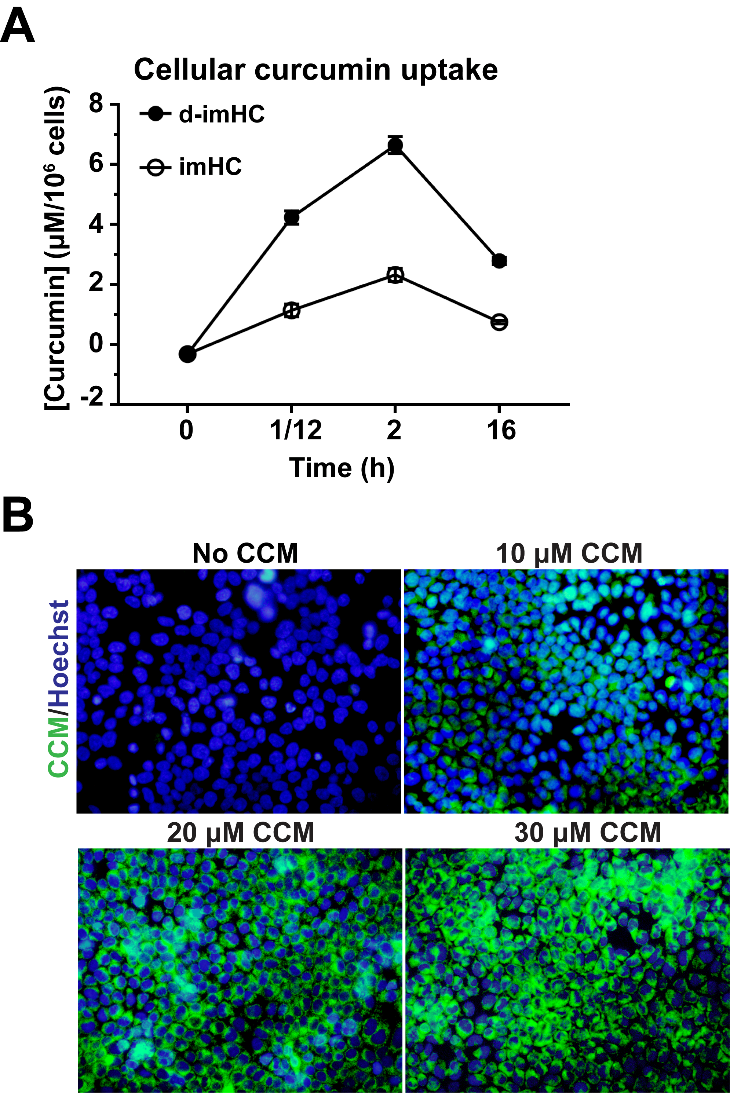


**Figure S3** CCM uptake into imHC and d-imHC was time-dependent and dose-dependent. Cultured cells were incubated with 30 µM CCM for 3 different durations (5 min, 2 h and 16 h). Cell lysate was prepared by sonication. Cellular level of CCM was analyzed in cell lysate using a fluorescent microplate reader and quantitated with a standard curve with RP^2^P = 0.9907 (A). For dose-dependent manner of CCM uptake, cells were treated with 10 - 30 µM CCM for 16 h. The fluorescent signal in lived cells was visualized under a fluorescent microscope. The nucleus was counterstained with Hoechst (B).


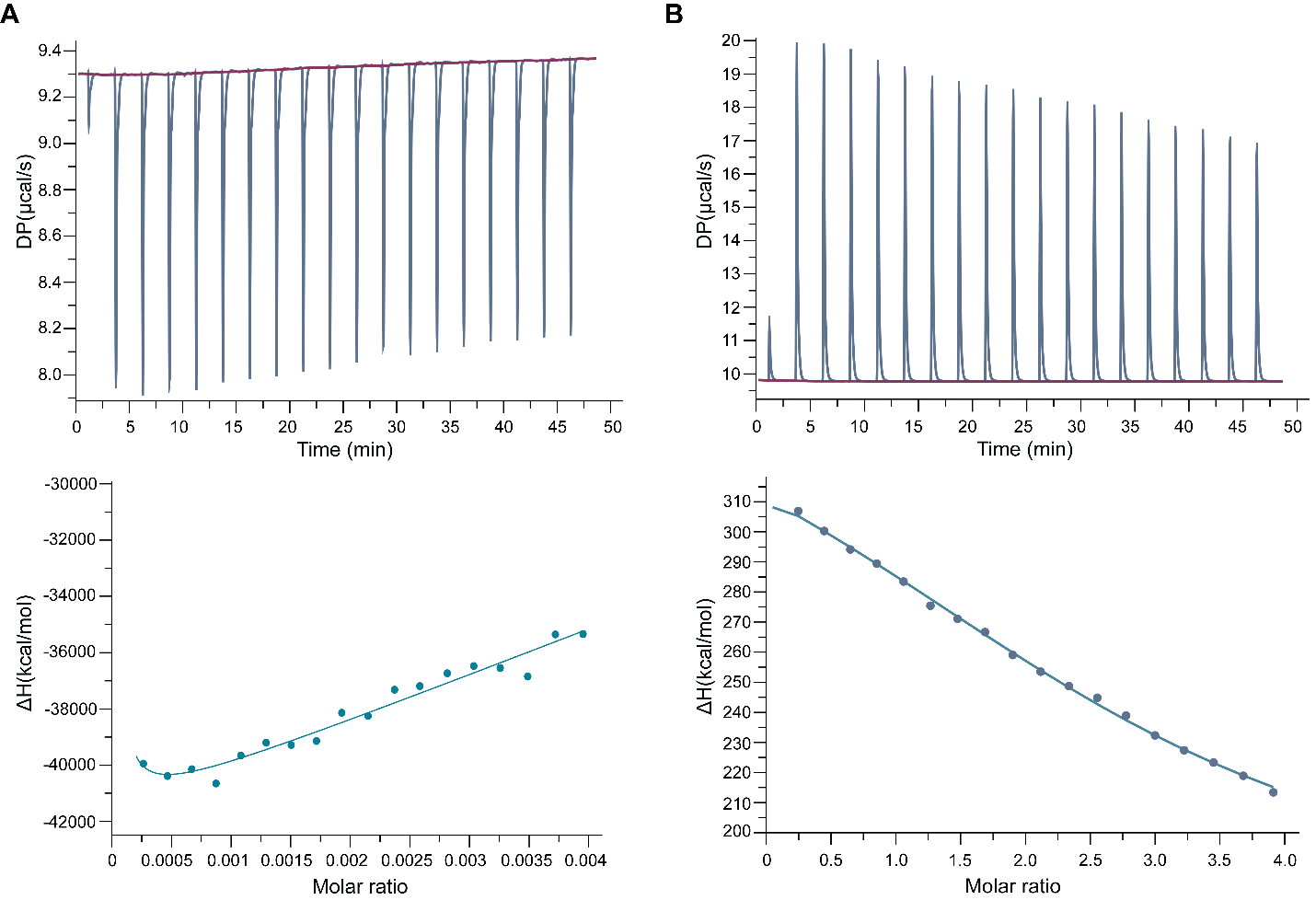


**Figure S4** The affinity of NTCP to individual chemical (TCA or CsA) was demonstrated using ITC. The ITC profile for the combination of NTCP with either TCA (A) or CsA (B) was generated from sequential injection of a chemical (150 nM TCA or 150 µM CsA) into the protein solution (15 μM of NTCP, pH 7.6, 25°C). The titration of raw data between differential power (DP, µcal/s) and time (min) were plotted. The enthalpy changes versus the molar ratios of ligands (injection) to protein (NTCP) were plotted. Data were fitted using a one-site binding model where the solid lines represented the best-fit results.

**
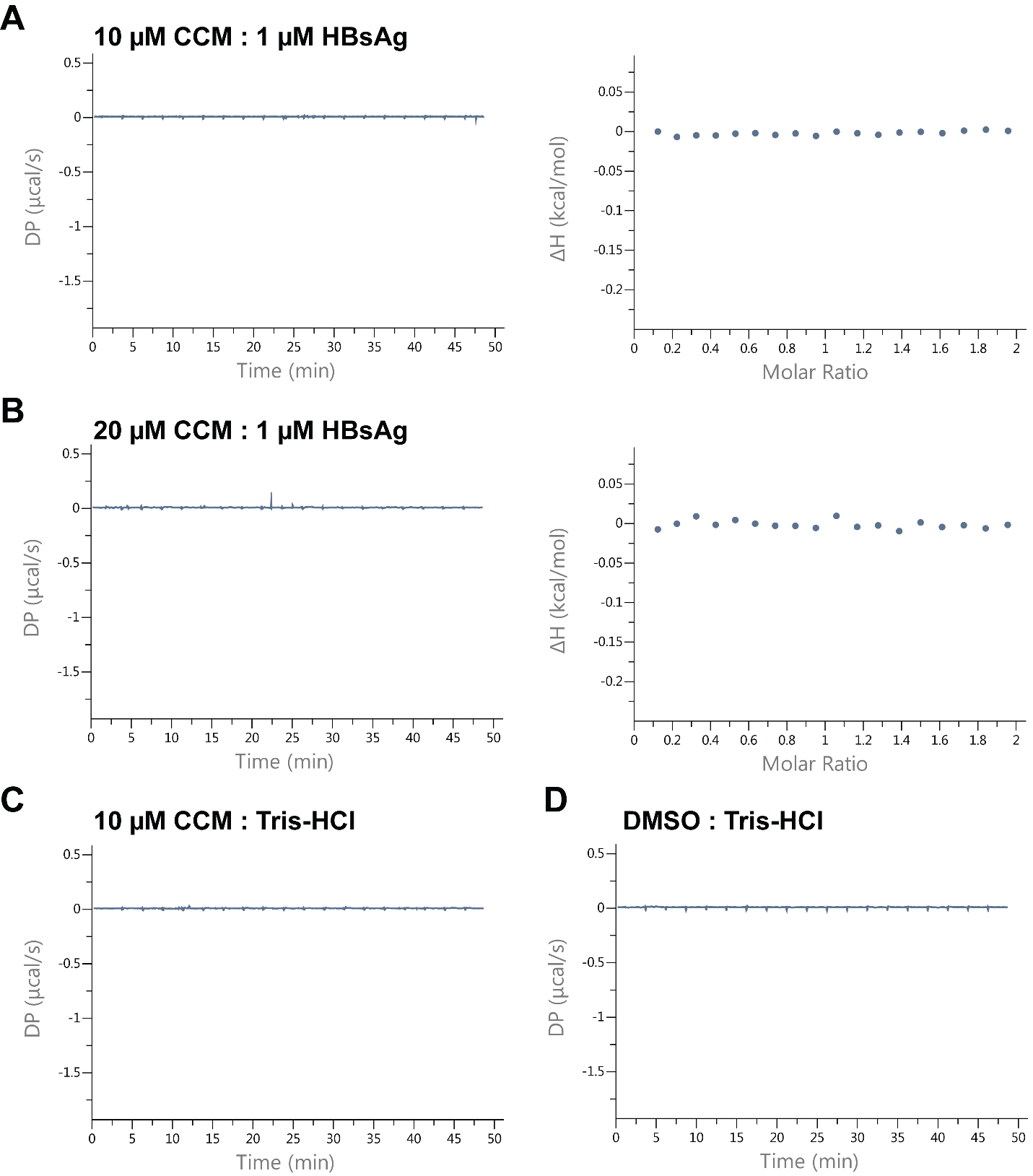
**

**Figure S5** CCM did not bind HBsAg. The association between CCM at 10 µM (A) or 20 µM (B) to 1 µM HBsAg was determined using ITC. The left panel exhibited the raw data for the sequential injection of ligand (CCM) into the protein solution. The right panel showed the enthalpy changes per mole of injected CCM plotted against the molar ratio of CCM to protein. Ligand (CCM)-buffer (C) and buffer-buffer (D) interactions served as negative controls.

**Table S1** Thermodynamic parameters resulting from the interaction between NTCP and individual chemical (CCM, TCA, or CsA) using ITC

| **Protein** | **ligand** | **Binding constant (**K_D_**) (M**P**^-1^**P**)** | **Enthalpy change (ΔH)**  **(kcal/mol)** | **Entropy change (ΔTΔS)**  **(kcal/mol)** | **Gibbs Free Energy change (ΔG)**  **(kcal/mol)** | **Type of binding** |
| --- | --- | --- | --- | --- | --- | --- |
| Human NTCP (SLA10A1) | CCM | 64.3×10P^-6^ | 80.0 | -85.7 | -5.72 | hydrophobic interection |
| Human NTCP (SLA10A1) | TCA | 2.56×10P^-10^ | -1.59 | -14.8 | -16.4 | hydrogen bond and hydrophobic interaction |
| Human NTCP (SLA10A1) | CsA | 9.29×10P^-6^ | -1 | -5.87 | -6.87 | Hydrogen bond and hydrophobic interaction |

**Supplementary Method 1:** Assessment of drug combination

Bliss independent model calculated the combined drug effect of different combinations (1). In brief, drug A at dose inhibits *(Y*R*_a_*R*)* percent of HBV replication while drug B at dose b inhibits *(Y*R*_b_*R*)* percent. When both were combined, the predicted additivity of probability theory (Y*P*R*_ab,P_*R*)* was calculated. The method compared the observed combination response (*Yo*) with the predicted combination response (*Yp*), which was obtained based on the assumption that there was no effect from drug-drug interactions.

*Y*R*_ab,P_*R *= Y*R*_a_*R *+ Y*R*_b_*R *˗ Y*R*_a_*R*Y*R*_b_*

The laboratory-observed percent HBV inhibition (*Y*R*_ab,_*RR*_O_*R*)* was compared with (YR*_ab,_*RR*_P_*R*)* that resulted in 3 probabilities.

| **Probability** | **Interactions** | **Type** |
| --- | --- | --- |
| *Y*R*_ab,O_*R *> Y*R*_ab,P_* | the observed combination was dramatically higher than expected response | Synergy |
| *Y*R*_ab,O_*R *= Y*R*_ab,P_* | the observed combination was equal to the expected response | Additivity |
| *Y*R*_ab,P_*R *< P*R*_ab,P_* | the observed combination was less than expected response. | Antagonism |

1. Zhao W, Sachsenmeier K, Zhang L, Sult E, Hollingsworth RE, Yang H. A New Bliss Independence Model to Analyze Drug Combination Data. Journal of Biomolecular Screening. 2014;19(5):817-821. doi:10.1177/1087057114521867
